# Supplementary material for: Territorial landscapes: incorporating density-dependence into wolf habitat selection studies
Source: R Soc Open Sci. 2019 Nov 20;6(11):190282. doi: 10.1098/rsos.190282 (PMC6894554; doi:10.1098/rsos.190282)
Supplement: Appendix S1 [file rsos190282supp1.pdf]

Appendix S1

A simulation of conceptual used & available resource distributions under assumptions of strong territoriality and population growth

Habitat selection is a dynamic behavioral process which can be influenced by many variables, including species territoriality. The effect of territoriality on temporally-varying, density dependent habitat selection trends has received little attention at the landscape scale. Specifically, the availability distribution is a key component of modern habitat selection models and can be constrained substantially at the population level when animals employ a preemptive site occupancy strategy. The implication is that the geographic availability of all habitat types shrinks in size as populations increase and vice versa. Depending on the degree of preference or avoidance of a particular habitat and the relative abundance of the habitat, the selection ratio can vary in ways that may be unanticipated. We simulated 4 scenarios of density (or occupancy) dependent habitat selection under the assumption that increases in occupancy led to constricted geographic availability of habitats. Depending on the initial habitat distribution and the nature of habitat use, the selection ratio increased, decreased, or remained constant over time. The change in the selection ratio is akin to a density dependent change in the (β) coefficients from modern habitat selection models and depends on the convergence or divergence of habitat use and availability distributions. For example, if the central tendencies of used and available habitat distributions diverge, the strength of habitat selection increases. Alternatively, the used and available distributions may converge, resulting in weaker selection, or move in parallel, resulting in constant selection.

Simulation approach

We explored the effect of changing habitat availability on continuous habitat coefficients for four scenarios of increasing population growth: S1 = a finite habitat type with strong selection and limited availability, S2 = an abundant habitat type which is selected for but not limited, S3 = a limited habitat type that is initially selected for but is increasingly substituted for by another habitat type as its availability declines, and S4 = an abundant habitat type with no initial selection, but becomes increasingly selected for as a substitute for another limited habitat type (e.g. the habitat in S3). For each scenario, we simulated occupancy on a 10 × 10 grid and specified 15 time steps. To model increases in occupancy, we specified a sigmoidal logistic growth function to represent an increase of proportion occupied from approximately 0.05 to 0.75 across the 15 unit time series. We used the 'scurve' function in R 3.2.2 package 'LS2Wstat' (Nunes et al. 2014) to extrapolate the values between 0.05 and 0.75 at time $t = (1, 2, \dots, 15)$. For each scenario, we specified a probability distribution function (PDF) for a continuous random variable representing an arbitrary habitat metric of interest, where the shape of the PDF was chosen depending on the scenario. For all cases, low values represented low quality habitat and vice versa. For example, a habitat with limited resources would have greatest density at values near or below 0, with greater values (i.e. upper tail of distribution) representing a limited supply of high quality habitat. Alternatively, a habitat with abundant resources would have greatest density at values above zero. We assumed constant territory size and occupancy in each example such that occupied territories could not overlap or change in size and once geographical units were occupied they remained so for all subsequent time steps.

Scenario 1 (S1)

In S1, we simulated dynamic habitat selection of a relatively limited, finite habitat type. Each geographical unit was assigned a habitat value $X_1 = (x_1, x_2, \dots, x_{100})$ based on a normally

distributed random variable with mean 0 and variance 1, i.e. $X_1 \sim N(0,1)$ (Fig. S1-1A). We specified the probability of an animal occupying a geographical unit *i* to be a sigmoidal function of X_1, with probability increasing as X_1 increased (Fig. S1-1B):

$$P(i=1) = \frac{1}{1+e^{-0.15x_1}} \tag{1}$$

where *i* = 1 indicates selection of unit *i*. The proportion occupied at each time unit similarly followed a sigmoidal function with a slope coefficient of 0.33: $N(t) = \frac{1}{1+e^{-0.33t}}$, where *N(t)* represented the number of units occupied at time *t*. We simulated occupancy by randomly sampling *n* units from the grid at each time step without replacement, where

$$n = \begin{cases} n(t) & \text{if } t=1 \\ n(t)-n(t-1) & \text{otherwise (t > 1)} \end{cases} \tag{2}$$

and probability of occupying unit *i* followed (1). At each time step we estimated the used ($f_u[X_1]$) and available ($f_a[X_1]$) distributions of the habitat X_1 by computing its mean in the occupied (used) and remaining unoccupied (available) units. We repeated the procedure 100 times to generate a sample of independent time series. We then graphically plotted the used and available habitats as a function of time, and computed the ratio of used to available habitat at each time step: $\beta(X_1,t) = \frac{\exp(f_{u,t}[X_1])}{\exp(f_{a,t}[X_1])}$. As a final step, we used a local polynomial regression smoother to fit a trend line to the simulated used and available distribution time series and selection ratios.

Scenario 2 (S2)

In S2, we simulated dynamic habitat selection of an abundant but used habitat type. Each geographical unit was assigned a habitat value $X_2 = (x_1, x_2, ..., x_{100})$, which was based on a Beta distribution, i.e. $X_2 \sim Beta(\alpha = 5, \beta = 1)$ (Fig. S1-2A). The probability of occupancy for unit *i* in

this case was specified similar to (1), with a slope parameter of 0.5 (instead of 0.15) and the range defined by X_2's range. Selection of X_2 was thus slightly weaker than that of X_1, with an increase in probability of ~ 0.9 across the range of X_2 (Fig. S1-2B). Occupancy was otherwise simulated as in (2), and the used and available distributions along with corresponding selection ratios were estimated similar to S1.

Scenario 3 (S3)

In S3, we simulated dynamic habitat selection of a limited habitat type, this time defining rules for a declining probability of occupancy as occupancy increased. A motivating example would be an animal that switches from a preferred food item to an alternative source, thereby substituting one habitat type for another. Geographical units were assigned habitat values $X_3 = (x_1, x_2, ..., x_{100})$, which were based on a Beta distribution with highest density at low values, i.e. $X_3 \sim Beta(\alpha = 1, \beta = 5)$ (Fig. S1-3A). To model declining habitat use, we defined an initial probability of occupancy similar to S1 and S2, defining a slope parameter of 0.75 and range defined by X_3's range, which corresponded to an initial probability of use increase of 100% across the full range of possible values (Fig. S1-3B). In this case, probability of use was a declining function of occupancy, modeled through the $\boldsymbol{\beta}$ coefficients of a generalized linear model (GLM) with a binomial response and logit link function. For example, the curve defining the initial relationship between X_3 and probability of use can be expressed as the linear model $\text{logit}(Y) = -7.5 + 15x + \varepsilon$, where x is the habitat value (in this case, X_3). We specified use of X_3 to decline to near zero at occupancy > 0.75, using a logistic function for β_0 and β_1 in the GLM

$$\beta_0 = \frac{-7.5}{1 + \exp\{(0.5 - x) / -0.06\}} \quad (3)$$

$$\beta_1 = \frac{15}{1 + \exp\{(0.5 - x)/-0.06\}} \quad (4)$$

where *x* was the proportion of units occupied. We used the ‘SSlogis’ function in R (Pinheiro et al. 2011) to estimate the scale and point of inflection parameters. Simulations proceeded as in S1 and S2, with estimation of used and available distributions along with the corresponding selection ratio.

Scenario 4 (S3)

S4 represents the substitute habitat type for S3, with initial selection near zero but increasing as occupancy increases. Geographical units were assigned habitat values $X_4 = (x_1, x_2, ..., x_{100})$, which were based on a Beta distribution with highest density at greater values, i.e. $X_4 \sim Beta(\alpha = 4, \beta = 2)$ (Fig. S1-4A). We defined an initial probability of use as in previous scenarios, this time with a slope parameter of 0.01 indicating very weak initial selection (Fig. S1-4B). In contrast to X_3, selection of X_4 was an increasing function of occupancy. Similar to S3, we specified an increase in β_0 and β_1 using logistic functions:

$$\beta_0 = \frac{-7.5}{1 + \exp\{(0.5 - x)/0.06\}} \quad (5)$$

$$\beta_1 = \frac{7.5}{1 + \exp\{(0.5 - x)/0.06\}} \quad (6)$$

where *x* was the proportion of units occupied. Contrary to the previous example, the scale parameter is positive, resulting in a decreasing effect on β. Simulations proceeded as in the other scenarios.

Results

Scenario 1 (S1)

Fig. S1-5 illustrates the simulated change in occupancy for one iteration of the 15-step time series, using S1 as an example. In S1, the animal exhibits strong selection for the habitat (greater habitat suitability in darker green), such that by the end of the time series the majority of remaining unoccupied units have unsuitable habitat values (Fig. S1-5, Fig. S1-6A). The results of all simulations are shown in Figure 6. In S1, the strong selection for habitat X_1 resulted in a decline in both the used ($f_u[X_1]$) and available ($f_a[X_1]$) distributions (Fig. S1-6A). However, in this example, the depletion of remaining available habitat was evident by the end of the time series, resulting in a decline in the value of available habitat that was steeper than that of the used habitat (Fig. S1-6A). As a result, the selection ratio (use proportional to availability) increased with increasing occupancy over time (Fig. S1-6B).

Scenario 2 (S2)

In S2, the animal used habitat at a rate that was nearly proportional to its availability (Figs. S1-2A, 2B, 6C). In this case, the used or available habitat distributions were both relatively static. As a result, the selection ratio also remained constant as occupancy increased (Fig. S1-6D).

Scenario 3 (S3)

S3 demonstrates declining selection over time, as the animal's probability of using an initially preferred habitat type decreases as that habitat type becomes less available. The distribution of used habitat decreases more rapidly than that of the available habitat distribution (Fig. S1-6E), resulting in a declining selection ratio with increasing occupancy (Fig. S1-6F).

Scenario 4 (S4)

In S4, the animal does not exhibit initial use of the habitat, but increases its use as occupancy increases. The result is an increasing used habitat distribution and a decreasing available habitat distribution (Fig. S1-4G), leading to an increasing selection ratio (Fig. S1-4H).

Synthesis

Simulated scenarios of habitat use, availability, and selection under strong assumptions of territoriality indicate that habitat selection can be temporally dynamic in ways that may be unexpected. Selection for a particular habitat can increase even when the habitat used by the individual or group declines over time due to density dependent saturation of high suitability habitat. In addition to providing ecologically relevant information that can often be overlooked, exploring temporal trends in habitat use and availability distributions will likely result in a more broadly informed analysis overall, and can reveal explanations for unexpected patterns in habitat selection. While this conclusion can also be applied to gregarious and social species, its significance is especially relevant to territorial species with population fluctuations resulting in range expansion or contraction over time. Modern evaluations of habitat suitability should recognize the potential for territoriality to constrain distributions of habitat availability, which are crucial for accurately assessing habitat selection.

References

Nunes, M. A., S. L. Taylor, and I. A. Eckley. 2014. A multiscale test of spatial stationarity for textured images in R. A peer-reviewed, open-access publication of the R Foundation for Statistical Computing:20.

Pinheiro, J. C., D. M. Bates, S. Debroy, D. Sarkar, and R Development Core Team. 2011. nlme: Linear and Nonlinear Mixed Effects Models.

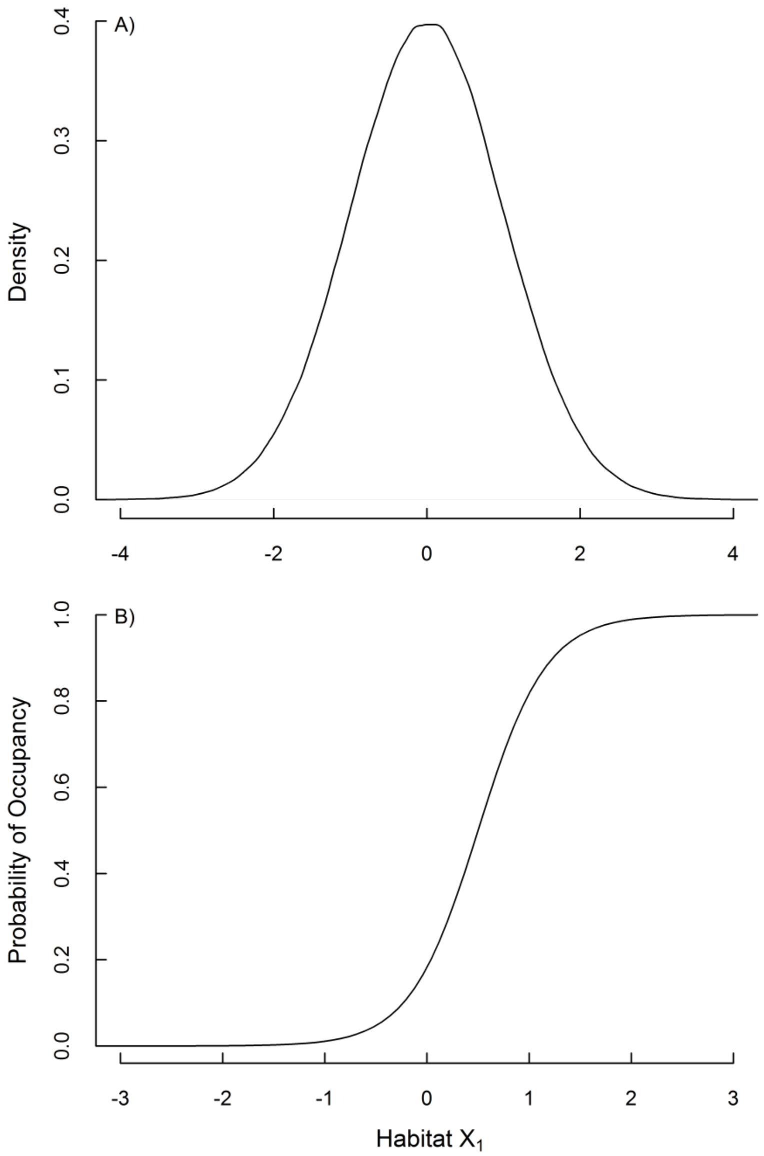

Figure S1-1. Hypothetical relationship between A) a simulated habitat and B) the probability of and individual or group occupying the habitat for Scenario 1 (S1). In S1, if the habitat is normally distributed ($X_1 \sim N$ (μ=0,σ=1)) and occupancy is related to larger habitat values, then suitable habitat is limited such that selection will deplete available habitat over time.

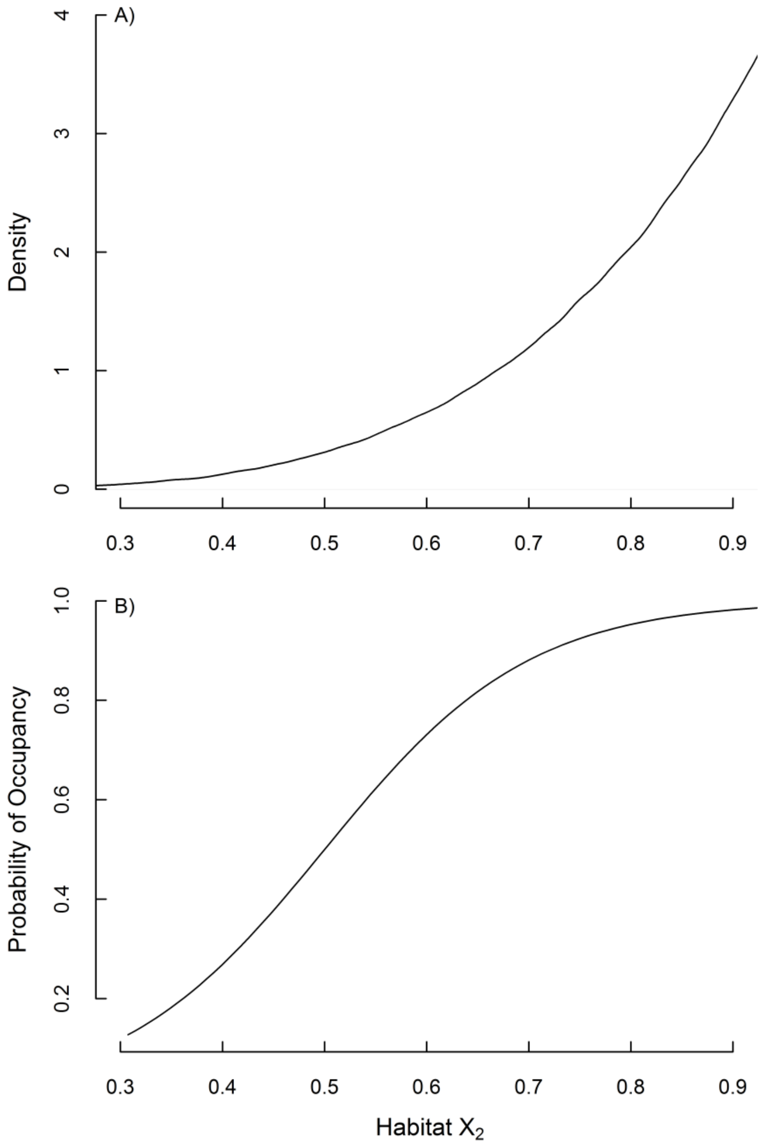

Figure S1-2. Hypothetical relationship between A) a simulated habitat and B) the probability of and individual or group occupying the habitat for Scenario 2 (S2). In S2, the habitat is Beta distributed ($X_2 \sim Beta(\alpha = 5, \beta = 1)$); although occupancy increases with increasing habitat values, the habitat type is more abundant and thus is less likely to be depleted.

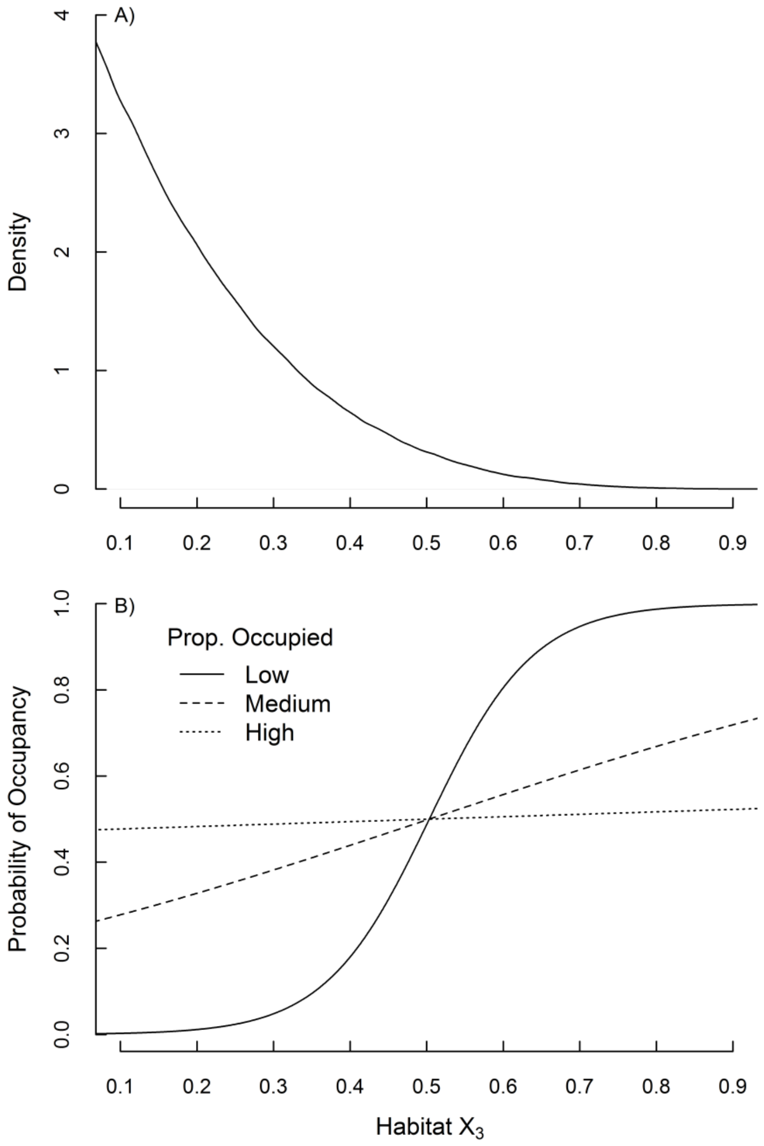

Figure S1-3. Hypothetical relationship between A) a simulated habitat and B) the probability of an individual or group occupying the habitat for Scenario 3 (S3). In S3, the habitat is Beta distributed ($X_3 \sim Beta(\alpha = 1, \beta = 5)$) and probability of occupancy depends initially on X_3 but declines as the proportion of the landscape occupied increases. This scenario may occur when an animal switches to an alternative habitat type as the initial habitat becomes less available.

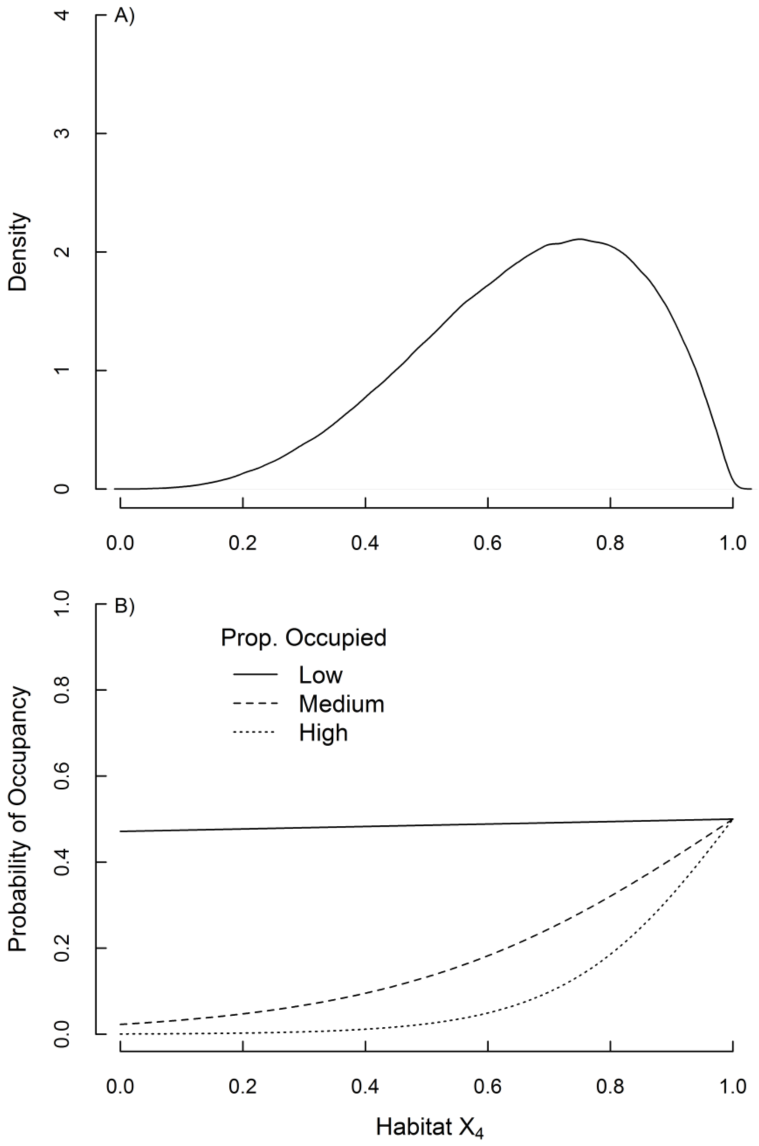

Figure S1-4. Hypothetical relationship between A) a simulated habitat and B) the probability of an individual or group occupying the habitat for Scenario 4 (S4). In S4, the habitat is Beta distributed ($X_4 \sim Beta(\alpha = 4, \beta = 2)$) with no initial relationship between X_4 and occupancy. However, as occupancy increases, habitat X_4 becomes increasingly important. This scenario may occur when an animal switches from an alternative habitat type.

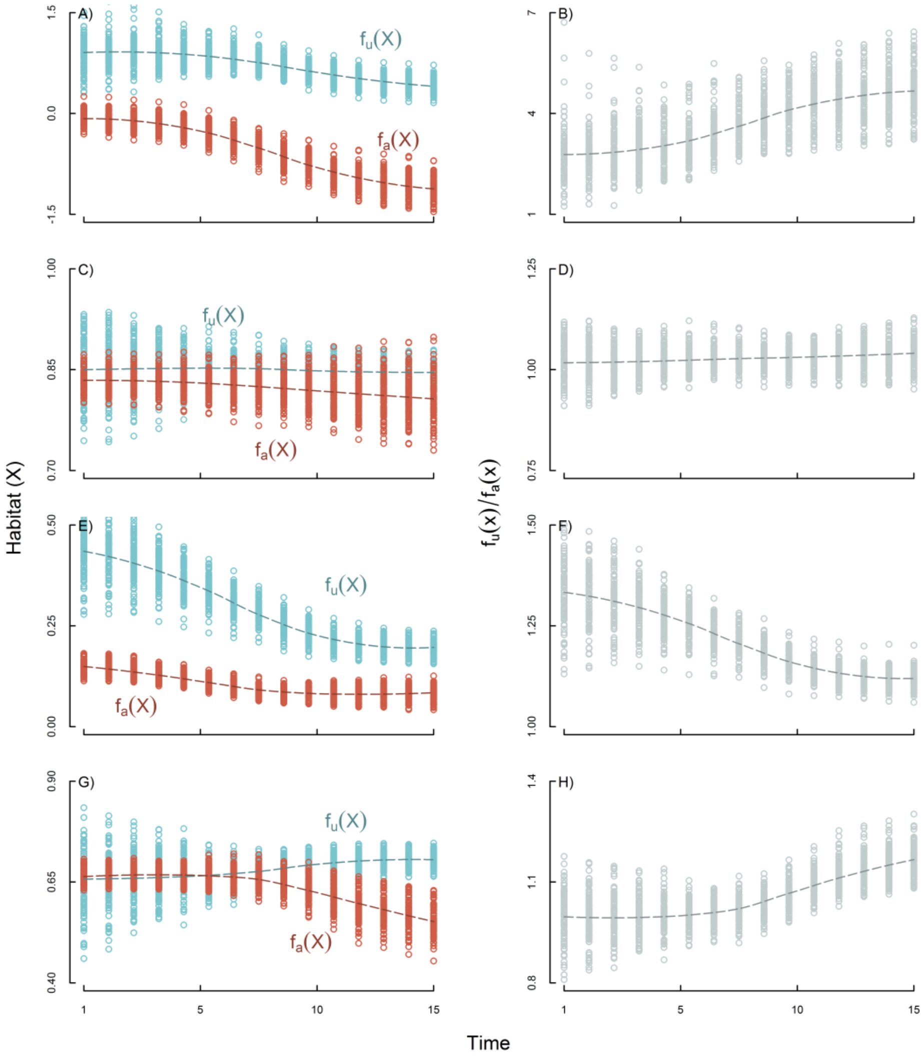

Figure S1-5. Results from simulations of used and available habitat (1st panel column; A,C,E,F) and corresponding selection ratios (2nd panel column; B,D,F,H) under assumptions of strong territoriality and increasing occupancy over time. Four hypothetical scenarios were evaluated in simulations, including an important, limited habitat (A,B), abundant but important habitat (C,D), limited substitutable habitat (E,F), and more abundant substitute habitat (G,H). Results show that the change in the selection ratio is dependent on convergence or divergence between used and available habitat distributions as occupancy increases over time.

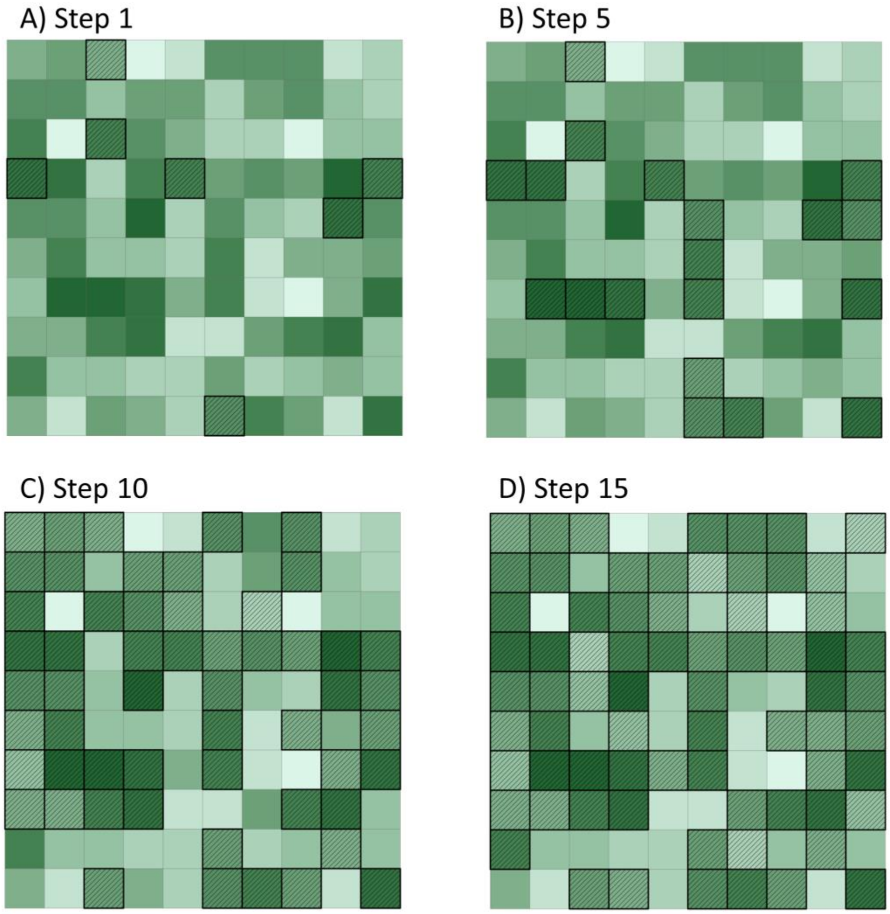

Figure S1-6. Demonstration of one simulation iteration of increasing occupancy over time from ~ 5% of units occupied to ~ 75% occupied for a limited but preferred habitat type. Bolded grid units with hash marks represent units that have become occupied at, e.g. time steps 1 (A), 5 (B), 10 (C), 15 (D), while darker shades indicate greater habitat suitability.
