# Supplementary material for: Territorial landscapes: incorporating density-dependence into wolf habitat selection studies
Source: R Soc Open Sci. 2019 Nov 20;6(11):190282. doi: 10.1098/rsos.190282 (PMC6894554; doi:10.1098/rsos.190282)
Supplement: Appendix S2 [file rsos190282supp2.pdf]

Appendix S2

Estimation of territory boundary and size in relation to type of method used

A combination of methods (ground tracking and aerial telemetry) were used to obtain a complete census of wolf pack presence and occupancy in this study. Here, we present the estimation of territory size in relation to those methods, to indicate any differences and associated potential bias associated with method. Methods used included Minimum Convex Polygon (MCP) of ground tracking locations for individual packs across time, annual kernel density estimate from telemetry when 30 locations were gathered during a year's time frame, and long-term average kernel density estimate when 30 locations were gathered for the pack over a 3-year time period. We report mean, median, and standard error of pack size estimates, and demonstrate the data in Fig. S2.

Method	Median	Mean (SE)
Telemetry (Annual KDE)	243 km^2	283 (172) km^2
Telemetry (Long-term KDE)	255 km^2	295 (141) km^2
MCP (Long-term track survey data)	247 km^2	259 (166) km^2

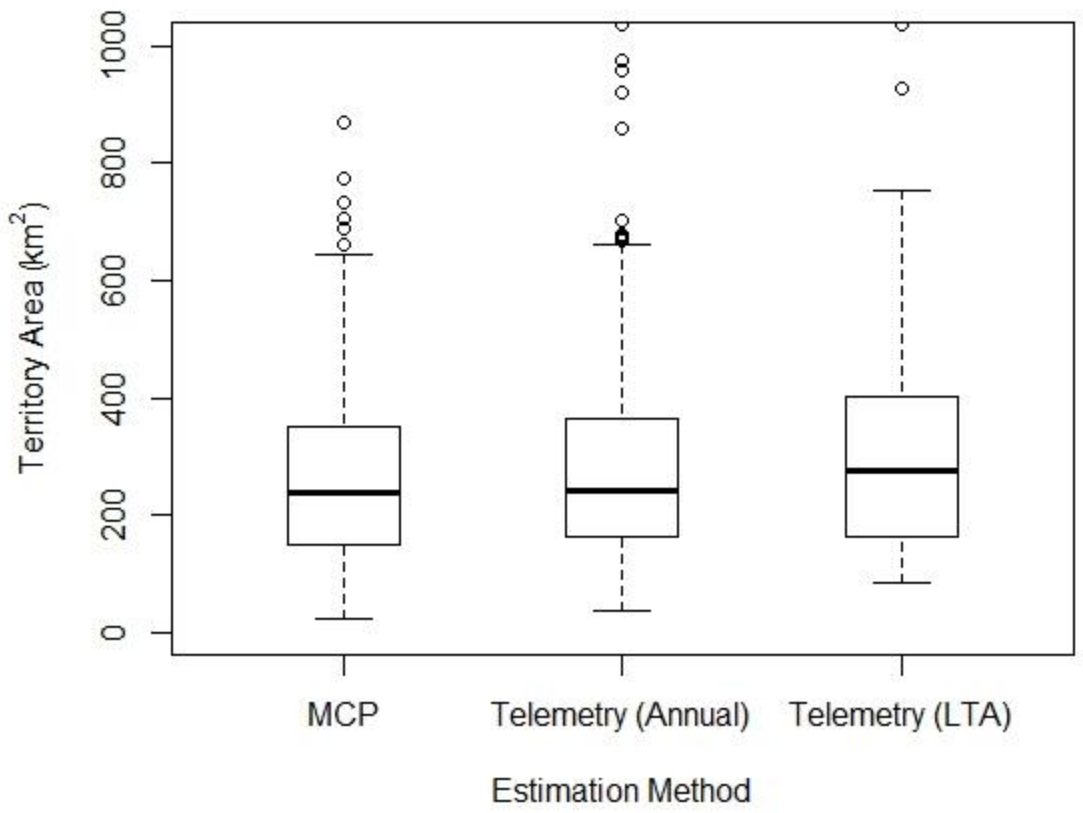

Figure S1. Distribution of estimated territory sizes (km^2) in relation to method of estimation. Methods included minimum convex polygon (MCP) of ground tracking data over time, annual estimate from kernel density estimator applied to telemetry observations, and long-term average based on kernel density estimator applied to telemetry observations.
