# Supplementary material for: Territorial landscapes: incorporating density-dependence into wolf habitat selection studies
Source: R Soc Open Sci. 2019 Nov 20;6(11):190282. doi: 10.1098/rsos.190282 (PMC6894554; doi:10.1098/rsos.190282)
Supplement: Appendix S3 [file rsos190282supp3.pdf]

Appendix S3

Candidate landscape variables in spatiotemporal habitat selection function (HSF) models, Michigan wolves 1994 – 2013

A. Index of Prey Availability (Resources)

Variable: Distance to Deer Wintering Complex

Source: MDNR (e.g. http://www.michigan.gov/dnr/0,4570,7-153-10363_10856_10905-339639--.html)

Original Unit: Distance to nearest DWC (km)

Description & Measurement: White-tailed deer (*Odocoileus virginianus*) are migratory throughout much of the UP due to harsh winter conditions, and congregate (i.e. "yard") in dense stands of primarily eastern hemlock (*Tsuga canadensis*) and northern white cedar (*Thuja occidentalis*) when snow depth exceeds approximately 30 cm (Shi *et al.* 2006; Witt *et al.* 2012; Murray, Webster & Bump 2013). Deer winter range was been mapped by state biologists as early as the 1930s, with surveys occurring every 10 – 20 years since. The most recent surveys occurred in 2005 and 2013. We used the maps from 2005 and 2013 to classify the study area as winter habitat (deer wintering complex, or DWC), or non-winter habitat. We generated the percent of landscape variable using a circular moving window (r = 4.02 km) to summarize the area mapped as DWC at each location. We generated distance to DWC (km) by creating a Euclidean distance raster surface in ArcGIS and subsequently calculating the average distance within the circular moving window across the study site. A continuous raster (30 m cell) was generated for the study area to represent these metrics. We assumed no significant change in DWC habitat throughout the study. Seasonal migration is a learned behavior and results in high fidelity to winter ranges, such that the same DWCs are repeatedly utilized year after year (Nelson 1998; Nelson, Mech & Frame 2004). We used an exponential decay function to transform the distance values to a more reasonable scale (0–1), where 1 indicated closest proximity,

Expected relationship with wolf space use: Positive (+), with respect to proximity. Deer wintering range is only about 15% of annual range in higher snowfall zones of the UP (Doepker *et al*. unpublished report). This represents a prey limitation that wolves must consider when establishing and defending territories. We expected wolf use to increase with increasing area of the landscape comprising DWC habitat and decreasing distance to DWC. This would correspond to a positive relationship with PPC1.

--

Variable: Annual and Long-term Average Snow Depth

Source: National Climate Data Center (http://www.ncdc.noaa.gov/)

Unit: Average Daily Snow Depth (cm), 1 Nov – 30 Mar (Annual); Cell-averages in cm for all 19-years of study (Long-term Average)

Description & Measurement: We downloaded daily snow depth data from the National Climatic Data Center (NCDC) for all weather stations on the study site. We calculated the mean winter snow depth at each station (1 Nov – 30 Mar) for each year in the study. We used Empirical Bayesian Kriging in ArcGIS (EBK; http://www.esri.com/news/arcuser/1012/empirical-byesian-kriging.html) to interpolate a raster surface for each year based on the weather station point data. Parameters for the EBK analysis included an output cell of 500 m, maximum number of points = 50, local model overlap = 2, simulated semivariograms = 50, and a standard circular neighborhood with radius = 150,000 m, maximum neighbors = 12, and minimum neighbors = 3. The resulting raster surface represented interannual and spatial variation in snow depths during the study. Final values were standardized around their mean for each study year prior to being fit in models.

Expected relationship with wolf space use: Conditional (+/-). In general, we expected habitat use to decrease with increasing snow depths (Houle et al. 2010, Uboni 2012), as habitat for prey became more limited (Potvin et al. 2005). However, greater snow depths may give wolves a hunting advantage, particularly during late winter when deer become more vulnerable and snow conditions become difficult to navigate (Vucetich et al. 2012). The use of DWCs may complicate this relationship; we expected greater selection of DWCs in areas (and winters) with deeper snow, as a response to more densely congregated prey.

B. Human Influence and Infrastructure (Risks)

Variable: % Agriculture

Source: National Land Cover Database (NLCD; http://www.mrlc.gov/)

Unit: Percentage of landscape comprising agricultural cover types (%)

Description & Measurement: Agricultural cover types were reclassified from NLCD products for years 1992, 2001, 2006, and 2011. The early years of the study were linked to results from the 1992 product (wolf biological years 1995-1997), and 2001 product (wolf biological years 1998-2003), while later years corresponded to the 2006 (wolf years 2004-2008) and 2011 products (wolf years 2009-2013). Agricultural cover types included pasture/hay, row crops, small grains, and fallow ground, and were assigned a value of 1, with all other cover types reclassified to Null values. The moving window was applied to calculate the percentage of landscape comprising agriculture at each location (30 m cell) in the study area. Final values were standardized around their mean prior to being fit in models.

Expected relationship with wolf space use: Negative (-). Agriculture typically represents mortality risk for wolves, and is negatively associated with pack persistence (Oakleaf et al. 2006, Mladenoff et al. 2009). Consequently, wolves are unlikely to select and maintain territories with a significant agricultural component, particularly when better habitat is available. We expected a negative relationship between wolf use and % agriculture, while recognizing that as wolf densities increased the relationship might change.

--

Variable: Distance to Major Road

Source: U.S. Census Bureau (http://www.census.gov/geo/maps-data/data/tiger.html)

Unit: Distance to nearest major road (km)

Description & Measurement: TIGER\Line roads were downloaded from the U.S. Census Bureau for the years 1990 and 2000-2014. We queried primary and secondary roads from the database for each year that the data were available. In the Upper Peninsula, these were almost entirely major highway routes. 1990 was removed from consideration because the classification scheme did not match the later years. Road coverages were similar in the 2000 data, however, so we used the 2000 file for the early study years. We calculated Euclidean distance to primary and secondary roads and applied the circular moving window to the resulting raster surface. For study years 2000-2013 we recalculated this metric every two years (i.e. 2002, 2004, … , 2012) to represent temporal changes in extent of roads. We transformed distances using an exponential decay function (0–1), where a value of 1 indicated closest distance.

Expected relationship with wolf space use: Negative (-), with respect to proximity. Many previous modeling efforts have indicated that wolf habitat is primarily limited by human activity and road densities. Some attention has been given to the type of road considered (Mladenoff et al. 1995, Oakleaf et al. 1995, Benson et al. 2014). While major roads are almost always avoided, lesser used roads can be utilized by wolves for traveling and territory marking (Lesmerises et al. 2012, Zimmerman et al. 2014). We split major and minor roads into separate classes to consider these possibilities. We expected wolf use to increase with increasing distance from major roads, because major roads are generally associated with higher levels of human activity and represent mortality risk.

--

Variable: Minor Road Density

Source: U.S. Census Bureau (see HWY)

Unit: Distance of Minor Roads / Unit Area (km / km^2)

Description & Measurement: TIGER\Line roads were described in ‘Distance to Major Road.’ For minor road densities, we queried local roads and trails from the TIGER\Line database. We then calculated minor road density within the moving window using the Line Density tool in ArcGIS. The temporal representation of these features was the same as in ‘Distance to Major Road.’

Expected relationship with wolf use: Negative (-). Traditionally, road densities have been considered a strong negative driver of wolf habitat quality. In general, wolf occurrence decreases with increases in human presence and disturbance, which often correlates with road density. However, in areas with relatively low human population density, wolves may select for areas with greater road densities than expected. This effect is context-specific, and depends on the

level of disturbance, such as current or recent logging activity (Houle et al. 2010, Lesmerises et al. 2012, 2013). Wolf use of these features tends to increase as human activity decreases (Hebblewhite and Merrill 2008). Logging occurs year-round in the UP and minor road densities are correlated with hunting camps and recreational activity, thus on average we expected a negative relationship between wolf use and minor road densities.

--

Variable: % Impervious Surface

Source: NLCD (http://www.mrlc.gov/index.php)

Unit: Percent of landscape comprising impervious surfaces (%)

Description & Measurement: In addition to the NLCD products, we also acquired the 2001, 2006, and 2011 Percent Developed Imperviousness product. In order to capture the best available temporal resolution for this feature, early study years were assigned to the 2001 product while later years (post-2002) were assigned to the 2006 and 2011 products (see description for '% Agriculture'). We summarized % impervious (focal mean) within the moving window described previously to create the index for human population density and infrastructure. Final values were standardized around their mean prior to being fit in models.

Expected relationship with wolf use: Negative (-). Imperviousness is used as an index of human activity and infrastructure, which wolves avoid if they can. We expected wolf use to decline rapidly as imperviousness increased.

--

Variable: % Protected Land

Source: USGS Protected Areas Database (http://gapanalysis.usgs.gov/padus/)

Unit: Percentage of landscape comprising public/protected land ownership

Description & Measurement: Protected areas in the UP of Michigan included National and State Forests, National Park Service land ownership, U.S. Fish and Wildlife Service, and various small tracts of land belonging to state or non-profit based conservancy projects or land trusts. We summarized the % landscape (focal mean) within the moving window to create an index of protected land where higher values represented lower potential for human development and disturbance. Final values were standardized around their mean prior to being fit in models.

C. Natural Features

Variable: % Open

Source: NLCD

Unit: Percent of landscape comprising open cover types (%)

Description & Measurement: We used NLCD products to calculate the percentage of open cover types occurring on the landscape. Open cover types and forested land were reclassified to a binary raster (1 = open), which was used to assess the proportion of open cover occurring within the moving window. Open cover types included grassland/herbaceous, pasture/hay, row crops, small grains, fallow ground, herbaceous/emergent wetlands, bare ground, and quarries, mines, or pits. The analysis was repeated for 1992, 2001, 2006, and 2011 NLCD products to represent land cover change during the study.

Expected relationship with wolf use: Negative (-). Wolves in northern forests often select cover types that are associated with prey access and ease of travel, and are negatively associated with human disturbances (Houle et al. 2010, Lesmerises et al. 2012, Kittle et al. 2015). In the UP, much of the open habitat is either associated with human disturbance such as logging and recreation, or agricultural land which represents risk to wolves. Wetlands are another open cover type, which may represent prey availability in the form of moose and beaver (Houle et al. 2010, Lesmerises et al. 2012), but moose are not abundant in the UP and beaver are, at best, a seasonal food source. We largely expected wolves in the UP to avoid open cover types, although the selection of this cover type is likely contextual. Alternatively, open areas may be used as hunting grounds, although this probably depends on the particular predator/prey system being studied (Kauffman et al. 2007). We considered seasonal and density dependent interactions to account for this possibility, as deep snow covers open areas in the winter, such that prey would likely only occur in these areas in the summer.

--

Variable: Open:Forested Edge Density

Source: NLCD

Unit: Distance of the open:forested linear feature / Unit Area (km / km^2)

Description & Measurement: We used NLCD products to create two binary rasters: one representing open cover types (see '% Open'), and one which included all forested cover types vs. other features. We defined the boundary between these two features as an edge, converted the boundary to line features in ArcGIS, and calculated the line density within the moving window described previously. The analysis was repeated for 1992, 2001, 2006, and 2011 NLCD products. Final values were standardized around their mean for each study year prior to being fit in models.

Expected relationship with wolf use: Positive (+). There are several reasons that wolf habitat use should be positively associated with edgy habitat. First, these features likely represent high quality foraging habitat for deer, particularly in summer, while also providing access to cover. In addition, linear features such as the transition zone between cover types may increase prey encounter rates and represent escape obstacles (Kaufmann et al. 2007, Houle et al. 2010, Lesmerises et al. 2012). Such features may also be useful as travel corridors and for marking territory boundaries. We expected habitat use to increase with increasing open:forested edge densities on our study site.

--

Variable: % Open Water & Wetlands

Source: NLCD

Unit: Percent of landscape comprising open water and wetlands

Description & Measurement: We used NLCD products to reclassify emergent/herbaceous wetlands and open water cover types. A binary raster was created for these cover types using methods described in '% Open.' We evaluated the percentage of landscape comprising open water and wetlands within the moving window. The analysis was repeated for the 1992, 2001, 2006, and 2011 NLCD products.

Expected relationship with wolf use: Negative (-). Water and wetlands may be indicative of potential alternative prey sources (beaver, moose), but wolves prey primarily on deer throughout the UP. Wolves selected against open water features, although shorelines were a preferred cover type (Kittle et al. 2015). In general, we expected wolf use to decline as the proportion of open water and wetlands increased.

--

Variable: Stream Density

Source: Michigan Geographic Data Library (http://www.mcgi.state.mi.us/mgdl/?rel=thext&action=thmname&cid=3&cat=MI+Geographic+Framework+Hydrography+%28v14a%29)

Unit: Distance of stream per unit area (km / km^2)

Description & Measurement: Hydrography files were downloaded from the Michigan Geographic Data Library. All streams and linear water features were selected from these data and clipped to the study area (FCC codes H3*– H4*). Linear stream features were converted to a 30 m density raster using the line density tool with 4.02 km radius.

Expected relationship with wolf use: Positive (+). Streams may represent higher prey availability, either via correlation with greater beaver densities (i.e. a seasonal/alternate prey source) or as preferred hunting territory (Kauffman et al. 2007, Lesmerises et al. 2012). We expected wolves to increase utilization with greater stream densities.

--

Variable: Elevation

Source: USGS National Map (http://nationalmap.gov/)

Unit: Meters above sea level (m)

Description & Measurement: We downloaded a 30 m DEM from the National Map and calculated mean elevation within the moving window described previously. Final values were standardized around their mean prior to being fit in models.

Expected relationship with wolf use: Negative (-), Quadratic (i.e. selection for intermediate elevation). Numerous studies have found a relationship between wolf territory use and elevation. While results are mixed, a common finding is that wolves do not utilize the highest elevations on the landscape, but also tend to select against lowlands on average (Lesmerises et al. 2012, Kittle et al. 2015). Thus, we expected selection for intermediate elevation on our study site (Milakovic et al. 2011). Although elevation does not exceed ~ 600 m in the UP, snowfall in winter tends to be greatest at higher elevations, and deer vacate these areas when snow exceeds ~ 30 cm.

--

Variable: Slope

Source: USGS DEM (see ELEV)

Unit: Degrees of slope (°)

Description & Measurement: We used the DEM described in 'Elevation' to compute degrees slope using ArcGIS, and calculated the mean slope within the moving window. Final values were standardized around their mean prior to being fit in models.

Expected relationship with wolf use: Negative (-), Quadratic (i.e. selection for intermediate elevation). We anticipated a similar response to slopes as that of elevation. The range of elevation on our study site was not large, but the topography in many areas was rugged, resulting in significant slopes and changes in elevation. We expected wolves to use areas with shallow or intermediate slopes on our study site. Steeper areas are more difficult for wolves to navigate and may be risky, while flatter terrain has been associated with wolf hunting behavior (Kauffman et al. 2007). Most studies find a negative relationship between wolf use and slope (Houle et al. 2010, Milakovic et al. 2011, Lesmerises et al. 2012).

--

Variable: Terrain ruggedness

Source: USGS DEM (see Elevation)

Unit: Index of terrain ruggedness

Description & Measurement: We used the DEM to compute the average terrain ruggedness index value within the moving window. The analysis was performed using the ArcGIS Geomorphometry and Gradient Metrics toolbox (Evans et al. 2014). The index measures topographic heterogeneity and is fully described in Evans et al. (2014).

--

Variable: Topographic radiation aspect index

Source: USGS DEM (see Elevation)

Unit: Index of heat load

Description & Measurement: We used the DEM to compute the average heat load index value within the moving window. The analysis was performed using the ArcGIS Geomorphometry and Gradient Metrics toolbox (Evans et al. 2014). The index measures potential for direct solar radiation and warmer temperatures based on a slope-aspect transformation; the method is fully described and referenced in Evans et al. (2014).

References

Benson, J. F., B. R. Patterson, and P. J. Mahoney. 2014. A protected area influences genotype-specific survival and the structure of a Canis hybrid zone. *Ecology* **95**:254-264.

Evans, J.S., Oakleaf, J., Cushman, S.A., and D. Theobald. (2014). http://evansmurphy.wix.com/evansspatial.

Frawley, B.J. 2010. Michigan deer harvest survey report 2009 seasons. Wildlife Report No. 3513. Michigan Department of Natural Resources and Environment. Available at https://www.michigan.gov/documents/dnr/report3513_327318_7.pdf.

Hebblewhite, M., and E. Merrill. 2008. Modelling wildlife-human relationships for social species with mixed-effects resource selection models. *Journal of Applied Ecology,* **45**:834-844.

Houle, M., D. Fortin, C. Dussault, R. Courtois, and J. P. Ouellet. 2010. Cumulative effects of forestry on habitat use by gray wolf (Canis lupus) in the boreal forest. *Landscape Ecology* **25**:419-433.

Kauffman, M. J., N. Varley, D. W. Smith, D. R. Stahler, D. R. MacNulty, and M. S. Boyce. 2007. Landscape heterogeneity shapes predation in a newly restored predator-prey system. *Ecology Letters* **10**:690-700.

Kittle, A. M., M. Anderson, T. Avgar, J. A. Baker, G. S. Brown, J. Hagens, E. Iwachewski, S. Moffatt, A. Mosser, B. R. Patterson, D. E. B. Reid, A. R. Rodgers, J. Shuter, G. M. Street, I. D. Thompson, L. M. Vander Vennen, and J. M. Fryxell. 2015. Wolves adapt

territory size, not pack size to local habitat quality. *Journal of Animal Ecology* **84**:1177-1186

Lele, S. R., E. H. Merrill, J. Keim, and M. S. Boyce. 2013. Selection, use, choice and occupancy: clarifying concepts in resource selection studies. *Journal of Animal Ecology* **82**:1183-1191.

Lesmerises, F., C. Dussault, and M. H. St-Laurent. 2012. Wolf habitat selection is shaped by human activities in a highly managed boreal forest. *Forest Ecology and Management* **276**:125-131.

Lesmerises, F., C. Dussault, and M. H. St-Laurent. 2013. Major roadwork impacts the space use behaviour of gray wolf. *Landscape and Urban Planning*, **112**:18-25.

Milakovic, B., K. L. Parker, D. D. Gustine, R. J. Lay, A. B. D. Walker, and M. P. Gillingham. 2011. Habitat selection by a focal predator (Canis lupus) in a multiprey ecosystem of the northern Rockies. *Journal of Mammalogy*, **92**:568-582.

Mladenoff, D. J., M. K. Clayton, S. D. Pratt, T. A. Sickley, and A. P. Wydeven. 2009. Change in Occupied Wolf Habitat in the Northern Great Lakes Region. Pages 119-138 in A. P. Wydeven, T. R. Deelen, and E. J. Heske, editors. Recovery of Gray Wolves in the Great Lakes Region of the United States. Springer New York.

Mladenoff, D. J., T. A. Sickley, R. G. Haight, and A. P. Wydeven. 1995. A regional landscape analysis and prediction of favorable gray wolf habitat in the Northern Great Lakes Region. *Conservation Biology*, **9**:279-294

Murray, B.D., Webster, C.R. & Bump, J.K. (2013) Broadening the ecological context of ungulate-ecosystem interactions: the importance of space, seasonality, and nitrogen. *Ecology,* **94,** 1317-1326.

Nelson, M.E. (1998) Development of migratory behavior in northern white-tailed deer. *Canadian Journal of Zoology-Revue Canadienne De Zoologie,* **76,** 426-432.

Nelson, M.E., Mech, L.D. & Frame, P.F. (2004) Tracking of white-tailed deer migration by global positioning system. *Journal of Mammalogy,* **85,** 505-510.

Oakleaf, J. K., D. L. Murray, J. R. Oakleaf, E. E. Bangs, C. M. Mack, D. W. Smith, J. A. Fontaine, M. D. Jimenez, T. J. Meier, and C. C. Niemeyer. 2006. Habitat selection by recolonizing wolves in the Northern Rocky Mountains of the United States. *Journal of Wildlife Management*, **70**:554-563.

Panzacchi, M., B. Van Moorter, O. Strand, L. E. Loe, and E. Reimers. 2015. Searching for the fundamental niche using individual-based habitat selection modelling across populations. *Ecography* **38**:659-669.

Potvin, M. J., T. D. Drummer, J. A. Vucetich, D. E. Beyer, R. O. Peterson, and J. H. Hammill. 2005. Monitoring and habitat analysis for wolves in upper Michigan. *Journal of Wildlife Management* **69**:1660-1669.

R Core Team. 2015. R: A language and environment for statistical computing. R Foundation for Statistical Computing Vienna, Austria.

Robin, X., N. Turck, A. Hainard, N. Tiberti, F. Lisacek, J. C. Sanchez, and M. Muller. 2011. pROC: an open-source package for R and S plus to analyze and compare ROC curves. *Bmc Bioinformatics* **12**.

Shi, H., Laurent, E.J., LeBouton, J., Racevskis, L., Hall, K.R., Donovan, M., Doepker, R.V., Walters, M.B., Lupi, F. & Liu, J.G. (2006) Local spatial modeling of white-tailed deer distribution. *Ecological Modelling,* **190,** 171-189.

Uboni, A. 2012. Wolf habitat selection at the territory level: seasonal and interannual variation and influence on reproductive success. PhD Dissertation. Michigan Technological University, Houghton, MI, USA.

Venables, W. N., and B. D. Ripley. 2002. Modern applied statistics with S. Springer Science & Business Media.

Vucetich, J.A., Huntzinger, B.A., Peterson, R.O., Vucetich, L.M., Hammill, J.H. & Beyer, D.E. (2012) Intra-seasonal variation in wolf Canis lupus kill rates. *Wildlife Biology*, **18,** 235-245.

Witt, J.C., Webster, C.R., Froese, R.E., Drummer, T.D. & Vucetich, J.A. (2012) Scale-dependent drivers of ungulate patch use along a temporal and spatial gradient of snow depth. *Canadian Journal of Zoology-Revue Canadienne De Zoologie*, **90,** 972-983.

Zimmermann, B., L. Nelson, P. Wabakken, H. Sand, and O. Liberg. 2014. Behavioral responses of wolves to roads: scale-dependent ambivalence. *Behavioral Ecology*, **25**,1353-1364.
