# Supplementary material for: Territorial landscapes: incorporating density-dependence into wolf habitat selection studies
Source: R Soc Open Sci. 2019 Nov 20;6(11):190282. doi: 10.1098/rsos.190282 (PMC6894554; doi:10.1098/rsos.190282)
Supplement: Appendix S4 [file rsos190282supp4.pdf]

Appendix S4

Preliminary model reduction to remove correlated and unnecessary predictor variable

Our model included a large number of correlated variables so we fit generalized linear models using penalized maximum likelihood to select a subset of predictors from the original set. We used the elastic net regularization penalty in R package 'glmnet,' which combines lasso and ridge regression methods to discard irrelevant predictors and shrink coefficients of correlated predictors toward each other (Friedman et al. 2010, Tibshirani et al. 2012). We set the parameter α to 0.9, which causes performance similar to the lasso but also manages erratic model behavior resulting from highly correlated variables (Friedman et al. 2010). To determine which effects to keep, we used cross-validation to estimate best fit for the tuning parameter λ, where the method for λ was set to choose the most regularized model with errors within one SE of the minimum mean cross-validated error (Friedman et al. 2010). To ensure consistency across the dataset, we ran a Monte Carlo simulation with 1000 iterations, randomly selecting 5000 records from our dataset, refitting the model with 'glmnet', and estimating λ via the cross-validation routine. Each time we recorded which variables had not been discarded, and calculated the overall proportion (p) of model fits that included each variable x. We dropped variables with $p < 0.5$, and included the rest in subsequent models.

References

Friedman, J., T. Hastie, and R. Tibshirani. 2010. Regularization paths for generalized linear models via coordinate descent. *Journal of Statistical Software* **33**:1.

Tibshirani, R., J. Bien, J. Friedman, T. Hastie, N. Simon, J. Taylor, and R. J. Tibshirani. 2012.

Strong rules for discarding predictors in lasso-type problems. *Journal of the Royal Statistical Society: Series B (Statistical Methodology)* **74**:245-266.
