# Supplementary material for: Territorial landscapes: incorporating density-dependence into wolf habitat selection studies
Source: R Soc Open Sci. 2019 Nov 20;6(11):190282. doi: 10.1098/rsos.190282 (PMC6894554; doi:10.1098/rsos.190282)
Supplement: Appendix S5 [file rsos190282supp5.pdf]

Appendix S5

Table S5. Estimation of gray wolf minimum population, pack size, and occupancy during a 19-year period of recovery in Michigan, USA. Population density (N/1000 km^2) was simply the total estimate of abundance divided by the entire study area (Upper Peninsula of Michigan, 42,991 km^2).

Year	N	N /1000 km^2	N (Packs)[a]	Pack size (mean)	Pack size (SE)	Area occupied (km^2)	Proportion of study area occupied
1995	80	1.86	27/32	2.74	0.86	5753	0.14
1996	116	2.70	23/41	3.33	1.81	6719	0.17
1997	112	2.61	31/50	2.89	1.08	9002	0.22
1998	140	3.26	39/46	3.14	1.39	9869	0.24
1999	174	4.05	55/67	3.02	1.32	13292	0.33
2000	216	5.02	65/71	3.21	1.46	16063	0.40
2001	249	5.79	73/76	3.49	2.06	15677	0.39
2002	278	6.47	70/76	4.29	2.34	15868	0.39
2003	321	7.47	79/88	4.56	2.54	15373	0.38
2004	360	8.37	89/92	4.60	2.76	17993	0.44
2005	405	9.42	98/103	4.59	2.54	20326	0.50
2006	434	10.10	99/109	4.65	2.71	19719	0.49
2007	509	11.84	111/114	4.91	2.50	22696	0.56
2008	520	12.10	122/129	4.40	2.66	23659	0.58
2009	577	13.42	120/125	5.18	3.40	22642	0.56
2010	557	12.96	128/131	5.06	3.16	23703	0.58

2011	687	15.98	135/137	5.07	2.81	25533	0.63
2012	NA		123	NA	NA	23335	0.57
2013	658	15.31	129/132	5.15	2.67	23967	0.59

[a] The first number indicates the number of wolf packs presumed present based on counts ≥ 2, obtained primarily from tracking data. The second number estimates additional packs (maximum pack estimate) based on telemetry data that suggested new formation of pack territories that were not otherwise counted.

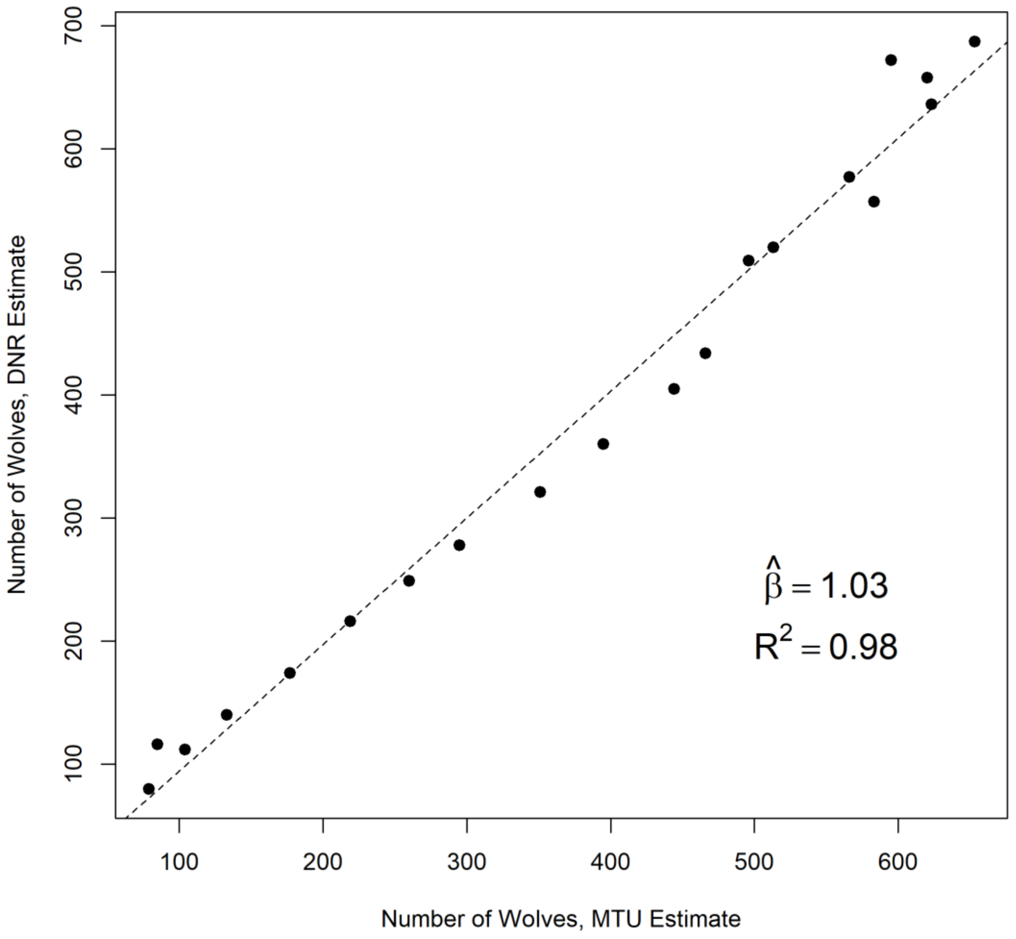

Figure S5. Comparison of wolf abundance estimated by the Michigan DNR to the estimates obtained by a modified method for evaluating spatial variation in wolf density, described in this

paper. Simple linear regression indicated strong correlation between the two methods (R^2 = 0.98).
